# Design of Garment Style Recommendation System Based on Interactive Genetic Algorithm

**DOI:** 10.1155/2022/9132165

**Published:** 2022-03-24

**Authors:** Yan Zhao

**Affiliations:** Fashion Art Design Department, Hubei Institute of Fine Art, Wuhan, Hubei 430250, China

## Abstract

Recommender systems provide users with product information and suggestions, which has gradually become an important research tool in e-commerce IT technology, which has attracted a lot of attention of researchers. Collaborative filtering recommendation technology has been the most successful recommendation technology so far, but there are two major problems—recommendation quality and scalability. At present, research at home and abroad mainly focuses on recommendation quality, and there is less discussion on scalability. The scalability problem is that as the size of the system increases, the response time of the system increases to a point where users cannot afford it. Existing solutions often result in a significant drop in recommendation quality while reducing recommendation response time. In this paper, the clustering analysis subsystem based on the genetic algorithm is innovatively introduced into the traditional collaborative filtering recommendation system, and its design and implementation are given. In addition, when obtaining the nearest neighbors, only the clustered users of the target user are searched, making it a collaborative filtering recommender system based on genetic clustering. The experimental results show that the response time of the traditional collaborative filtering recommender system increases linearly with the increase in the number of users while the response time of the collaborative filtering recommender system based on genetic clustering remains unchanged with the increase in the number of users. On the other hand, the recommendation quality of the collaborative filtering recommender system based on genetic clustering is basically not degraded compared with that of the traditional collaborative filtering recommender system. Therefore, the collaborative filtering recommender system based on genetic clustering can effectively solve the scalability problem of the collaborative filtering recommender system.

## 1. Introduction

The style of clothing has always been a vague and difficult-to-quantify concept; in the eyes of different people, the same style of clothing may be viewed differently, which is related to people's preferences, psychological and emotional state at that time, and many other factors. The description of clothing style is “casual” and “elegant,” but what counts as “casual” and what counts as “elegant?” All have their own views, which are based on people's personalized demand for clothing style. If we can grasp people's preferences and master their personalized needs, we can meet their needs in clothing recommendation and design clothing for them better.

Nowadays, personalized recommendation technology has attracted a lot of attention. At present, almost all large e-commerce systems, such as Amazon, CDNow, and Netflix, are using various forms of recommendation systems to varying degrees. Some information providers put forward the concept of personalized information service. Yahoo, physician information customizers, and fish wrap systems offer personalized information search. For example, in Yahoo, when users log in for the first time, the website will provide a series of personal interest options, which users can fill in and submit. In future logins, the site automatically pushes relevant information to the user first, which can be said to have a certain degree of personalization. The application of personalized service in China is far less extensive than that in Europe and America. The research pace of personalized recommendation should lead that of application. However, compared with Europe and the United States, China is still catching up in the application of considerable potential. Typical research achievements include the collaborative filtering distributed optimization algorithm proposed by Shanghai Jiao Tong University, real-time personalized recommendation based on the classification method by the Institute of Computing Technology of the Chinese Academy of Sciences, and personalized recommendation based on content filtering by Tsinghua University. At present, there are very few research studies on personalized clothing recommendation based on people's preferences.

In recent years, personalized garment customization has become an important part of garment production and sales. However, the existing MTM technology has complex software operation and requires a high threshold of professional knowledge, which are barriers to meeting the needs of customer-led design [1]. How to construct an easy-to-use and convenient interactive design system of clothing style has become a research hotspot.

Personalized clothing design is based on parts [2–4]. The customer-driven rapid three-dimensional clothing modeling method is used to input the designed clothing component models to build the component library of virtual clothing, which is convenient for users to quickly obtain rich clothing virtual simulation models from samples with different combinations [5, 6]. A reasonable style recommendation system can quickly search and accurately display data based on customer preference from a pool of data. At present, there are garment recommendation systems based on improved Bayesian networks, garment recommendation systems based on ontology [7], garment recommendation systems based on mold paste set theory [8], intelligent fashion recommendation systems based on key point garment style recognition [9], etc. Meanwhile, some scholars introduced interactive genetic algorithms in the optimization algorithm incorporated into style design and recommendation systems [10]. Lim et al. [11] proposed a new coding scheme that could integrate the professional knowledge in the clothing field into a genotype and developed a more practical auxiliary system for women's wear design. Nagamachi [2] proposed a garment consulting and simulation system based on the interactive genetic algorithm to help designers obtain the best matching scheme of product components and decorative patterns.

The IGA also has shortcomings, namely, the number of users in an interactive process when evaluating each individual is high, the high number of individual evolutions takes a long time, and the user can easily get tired, especially when there is a difference in a generation of four individuals; because of their relative advantages and disadvantages in assessment, the psychological pressure is huge, so they can be easily affected by fatigue. In order to solve the problem of user fatigue, the radial basis network function approximation ability of the neural network and the characteristics of good representation of the best individual in each generation of genetic operation are combined. This paper proposes that the best individual in each generation of genetic operation should be taken as the hidden node center value of the radial basis network. The data center, expansion constant, and output weight of the Gaussian kernel function are obtained by using the similarity distance value and the k-means method. In this paper, the implementation of a clothing design system based on this algorithm is given, and its effectiveness is proved.

In order to improve the recommendation accuracy of the traditional ontology algorithm, the recommendation process is improved, and the genetic algorithm of machine learning is combined with fuzzy ontology. This section describes the concepts and background information required in this article. The existing recommendation algorithms are mainly divided into two categories, namely, content-based algorithms and collaborative filtering algorithms. The former uses content-based technology to produce recommendations, while the latter uses feedback, ratings, user preferences, historical data, and so on to produce recommendations. More recently, advanced computer technology has facilitated the spread of machine learning in many applications, including recommendation systems. Machine learning simulates human judgment, decision making. and subjective evaluation of choice in recommendation systems. In digital libraries, machine learning is applied to search books, design book structures according to learners' preferences, recommend books according to learners' behaviors, interests, and habits, evaluate reading materials, classify and outline books, adjust books according to students' learning styles, and so on.

However, the above-mentioned methods have a common limitation, that is, when analyzing the opinions of educators or students, only the clear values are considered, but the fuzzy values are not analyzed. However, the textual and online data used to present the teaching and learning preferences of teachers and students are often inaccurate and sometimes mixed with inconsistent and irrelevant personal subjective judgments. The traditional recommendation method cannot solve the problem of personal subjective judgment. Therefore, fuzzy ontology is proposed to solve the problems of uncertainty in the category of books in book recommendation and the subjective judgment of users in choosing books. When the ambiguity is small, it indicates that the user is more confident and certain about the selected information. In this paper, a new framework of the recommendatory system based on correlation semantics is proposed, which can describe the features of recommendatory books with corresponding weights so as to convert the features into characters in the relational method. The weights of related features were optimized by the genetic algorithm.

The traditional genetic algorithm can only solve the system optimization problem with the explicit representation of the performance index because the fitness function expressed by an explicit expression is needed to evaluate the fitness of evolved individuals. However, many practical system optimization problems, such as image generation, music creation, industrial design, data mining, and other performance indicators, are often difficult to express by explicit functions, and the traditional genetic algorithm cannot solve these problems. This method is called the interactive genetic algorithm (IGA) and is expected to effectively solve the above-mentioned system optimization problems by combining human intuitive evaluation with the traditional evolutionary mechanism as the fitness of the evolutionary individual.

Compared with the traditional genetic algorithm, the interactive genetic algorithm has the following three characteristics: (1) humans do not evaluate the phenotype of evolved individuals but directly evaluate the system output determined by the phenotype. (2) People may give the same fitness value for different evolutionary individuals at different evolutionary stages and may give different fitness values for the same evolutionary individuals at different evolutionary stages. However, the existing research results show that the interactive genetic algorithm is robust to the “noise” generated because of the above-mentioned reasons. (3) The optimization solution of the interactive genetic algorithm is a region—optimization region—rather than one or more discrete points like the traditional genetic algorithm, but it has met the requirements for image generation, music creation, data mining, and complex system optimization.

The research on the interactive genetic algorithm started with Dawkins' work in 1986, and then there appeared two research directions: one is the field of artificial life, and the second is to focus on personalized or sensory systems, that is, systems with humanized information processing and human relevance. Currently, interactive genetic algorithms are mainly applied in the following areas: graphic image processing, speech processing and prosody control, face image generation, control and robotics, geophysical science, art education, and so on.

Human fatigue is the main problem of the interactive genetic algorithm because it needs humans to evaluate fitness. In order to solve this problem, people use small evolutionary generation and a small population size, but this will reduce the search of solution space, which is not conducive to find the optimal solution. To solve this problem, a cooperative algorithm is introduced in this paper to realize the simultaneous evolution of multiple populations and enlarge the search space, which is beneficial to the convergence of the algorithm.

The cooperative interactive genetic algorithm can be divided into two parts: one is the cooperative genetic algorithm, and the other is the interactive genetic algorithm. In the collaborative operation, information of other users is needed to replace the individual with the smallest adaptive value in the current generation, and this operation should be combined with interactive evolution operation. Specifically, after a generation of individuals goes through selection, crossover, and mutation to produce a new generation of individuals, users rate their phenotypes for fitness. There must be a minimum fitness after evaluation. Then a collaborative operation is carried out to replace the individual with the least adaptive value with the individual with the highest adaptive value of a generation of other users.

The two criteria for evaluating recommendation systems are recommendation quality and extensibility. Recommendation quality measures whether the recommendation results are good or bad, and the measurement standards mainly include the statistical accuracy measurement method and the decision support accuracy measurement method [12]. Scalability measures whether the system can work well in the case of continuous expansion of the scale of the system, and scalability criteria mainly refer to whether the response time can be maintained in the range of user tolerance in the case of continuous expansion of the system. The goal of recommendation system research is to design and implement high-quality and fast response collaborative filtering recommendation systems. It is found that the two major problems of collaborative filtering recommendation technology are recommendation quality and scalability. Improving recommendation quality and improving extensibility are essentially antithetical to each other. Research at home and abroad, especially in China, mainly focuses on recommendation quality, but there are few discussions on extensibility. The recommendation quality problem is mainly caused by data sparsity. The sparsity problem means that users only rate a very small part of all items, and most of the items have no rating data. The project scoring data matrix is extremely sparse, which leads to the decline in recommendation quality. In terms of sparsity, the authors in [13] proposed that the default value should be used to fill the 0 value of the user item-score data matrix, and a default value that is commonly used is the mean value. The main problem is that items that the user has not rated cannot all take the same default value. To calculate the similarity between projects in literature [14], by users of similar project score predictions for evaluation of the project, a more common score between user programs is made, which can effectively solve the user ratings under the extreme condition where data are sparse, which is the shortage of the traditional similarity measure method in calculating the nearest neighbors of target user more accurately.

For design of clothing of components, this paper establish a Polo shirt style and texture design component database and introduces an interactive genetic algorithm based on part similarity optimization; a personalized Polo unlined upper garment design recommendation system is set up, hoping to reduce the design professional threshold with more efficacy and convenience for the Polo unlined upper garment in personality customization based on user preference.

The written contributions of the paper are as follows:

1. In this paper, the cluster analysis subsystem based on the genetic algorithm is innovatively introduced into the traditional collaborative filtering recommendation system, and its design and implementation are given.
2. When obtaining the nearest neighbors, only the clustered users of the target user are searched, making it a collaborative filtering recommendation system based on genetic clustering.
3. The recommendation quality of the collaborative filtering recommender system based on genetic clustering basically does not decrease compared with that of the traditional collaborative filtering recommender system. Therefore, the collaborative filtering recommender system based on genetic clustering can effectively solve the scalability problem of the collaborative filtering recommender system.

The chapters for the remainder of the thesis are organized as follows:

Section 2 discusses the decomposition and modularization of clothing style elements, Section 3 discusses the genetic clustering subsystem, Section 4 discusses experimental results and analysis, and Section 5 summarizes the full text.

## 2. Decomposition and Modularization of Clothing Style Elements

The modular design of clothing style is based on the analysis of the style structure of clothing products and gradually realizes the division of module series and clothing series according to the needs of customers for module selection and module combination, forming clothing products. Clothing modular design is a customer-oriented design method, which can realize mass customization and agile production. However, in the interactive design system, it is necessary to store a large amount of clothing style module information and various clothing types for consumers to choose, so it is necessary to plan and design clothing types and style elements to complete the basic requirements of modular design.

### 2.1. Analysis of Clothing Types and Style Characteristics

The determination of clothing type is the beginning of any clothing design, just as the customer first determines whether the clothing type he or she wants to buy is a jacket or a bottom or a T-shirt or a shirt. However, according to the different classification bases, there are many different classification methods of clothing. However, in the design and purchase of daily clothing, these classification methods are not parallel, but longitudinal and in-depth, with a hierarchical cross structure. Therefore, the following is a detailed classification of clothing according to these classification elements.

The classification of clothing consists of two layers. The first layer is the classification of clothing categories, that is, the classification of different clusters after clustering on the basis of the similarity of clothing categories, such as outerwear clustering and clustering of pants. There are great differences among major categories of clothing, and a few of them can be used universally. The second layer is the internal division of clothing categories, that is, the division is made according to the differences in some aspects after the classification of clothing. For example, a short skirt and a long skirt are both skirts, but they are different in length, so they are divided into two types of skirts. Garment classification is the first layer of the meaning of long-term development of the gradually formed clothing classification. Common humans focus on the clothing design, with no effect of specification of clothing categories. Costume design often refers to the design of the second layer classification meaning of clothing: the detailed clothing design; hence, this article is on the basis of classification of the clothing categories. The second layer of clothing classification is studied in detail. First, clothing categories are classified and summarized according to the literature and network resources; then in-depth interviews are conducted with clothing experts to improve the clothing classification.

### 2.2. Types of Clothing

The classification of clothing includes factors such as age, gender, dressing season, dressing function, dressing part style characteristics, etc. Gender and age are the fundamental basis for classifying clothing. From the perspective of gender, clothing includes men's clothing, women's clothing, and neutral clothing. From the perspective of age, clothing includes clothing for 0- to 1-year-old baby, 2- to 5-year-old children, 6- to 11-year-old children, 12- to 17-year-old youth, 18- to 30-year-old youth, 31- to 50-year-old adult, and people older than 50 years.

According to the dressing season, clothing can be divided into spring and autumn clothing, summer clothing, winter clothing, and clothing for four seasons. Seasonal clothing refers to clothing that can be worn in any season. For example, ordinary shirts can be worn outside in summer or as under coats in winter as close-fitting clothing. Clothing categories divided according to the season have a great relationship with the thickness of the clothing surface. Thick clothing is mainly used in winter, thin clothing is mainly used in summer, and medium thickness clothing has a wide range of applicability and can be worn throughout the year.

**Figure 1:**
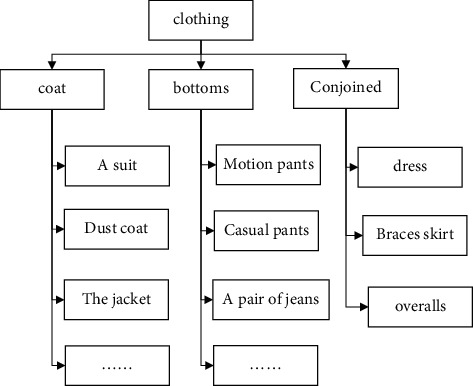
Hierarchical diagram of clothing classification.

There are many ways to classify clothing, and some of the less common ways include the purpose of wearing clothing, appearance of clothing, regional climate, nationality, production method, dressing method, dressing state, composition form, history, and so on. These classifications are rarely used and are therefore not explained in detail. In fashion design, the first is to determine the type of clothing according to the user's gender and age, and then refined style design is designed according to consumer demand or according to the use of the clothing function. The clothing subdivision applied to the system is shown in Figure 1, which is divided into three layers. The second layer is divided into top-, bottom-, and one-piece according to the position of the clothing. The third layer is subdivided according to the function of the clothing, covering almost all kinds of clothing.

### 2.3. Style Features of Clothing Jackets

It reflects the basic characteristics of clothing as a whole, and its types and quantities are limited. The silhouette of clothing is the most direct manifestation of the fashion style of clothing. It has a great relationship with the waist style characteristics and length and also reflects the personality and hobbies of the wearer. Therefore, the silhouette is included in the analysis of variable features as one of the style characteristics in this paper. The commonly used methods to represent the garment profile include letter representation, geometric representation, body representation, and description. In this paper, the clothing profile is divided into A, H, X, V, and O by letter representation. Type A creates a modest trapezoid impression by contracting the shoulders and exaggerating the hem. This silhouette of women's wear can add a lively, natural, youthful, flowing sense of strong, dynamic characteristics in the lady-like taste. The H model is a flat shoulder, non-tightening waist, tube form lower swing, weakened at the shoulder and thereby the bosom, waist, and buttocks. The width difference between 3 persons covered up the bloated feeling of the waist. The outer outline resembles the capital English letter H and gets its name. The silhouette clothing has the characteristics of being slim, simple, loose, comfortable to wear, and relaxed and having a stable style. The X shape is based on the body shape of people to shape the exaggerated shoulders and skirt hem and tighten the waist so that the overall appearance shows the upper and lower parts loosely exaggerated and narrow in the middle, as in the shape of the letter X. Because it is consistent with the beautiful curve of the female body, women's dress with soft, beautiful, sexy, and feminine characteristics in the classic style and ladies' style is used more. The V-shape is shaped like a capital letter V by enlarging the shoulders and shrinking the hem so that the shape is extended up and to both sides. This silhouette of clothing is quite generous, free, easy, and more masculine and has other characteristics. Type O is often designed with off shoulders to weaken the straight shape of the shoulders. By expanding the waist size of garments and shrinking the bottom edge, it forms a shape similar to that of a silkworm cocoon, which is usually called a cocoon type.

The design details of clothing refer to the design composition of different parts of clothing and are the specific combination forms of clothing composition whose types and quantities are infinite. For example, the garment jacket can be divided into collar, sleeve, and garment body, and the collar can be divided into collarless, standing collar, lapel collar, mottled collar, special collar, etc., each of which can have a variety of design examples.

The collar style is mainly refers to the style of the collar, including collarless, flat collar, standing collar, lapel collar, flapping collar, and hooded collar. These design classifications can have a variety of designs according to the different quantities of the edge, for example, in the plan that does not have a collar, the edge line of the collar ministry can be circular, squared, V-shaped, a polygon, irregular shaped, and so on.

### 2.4. Product Family Structure Network

**Figure 2:**
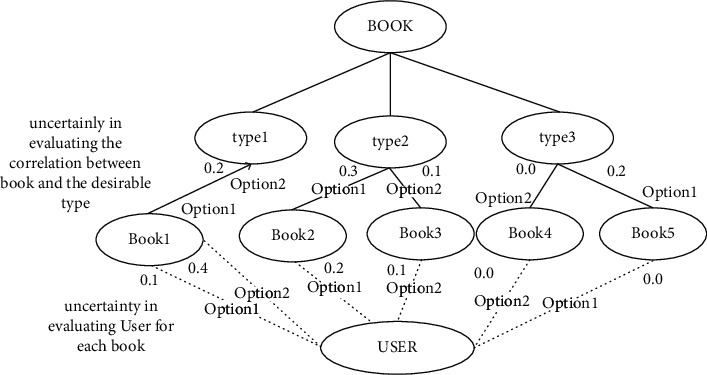
Fuzzy ontology representation involving the uncertainty of user and book concept evaluation.

A weighted directed network topology is constructed. The node can be either the whole garment or a single module in the garment product. The directed edge represents the subordinate relationship between the garment and the component module, and the direction is from the upper level to the next level of its membership.  Figure 2 shows a product family composed of two clothing items. Node C in the figure (*I I* = 1, 2,…, *n*) represents individual clothing style design, node *S*_*j*_ (*j* = 1, 2,…, *m*) represents the clustering of garment component modules, S_jk_ represents the specific module design, *E*_*i*_ represents the attribute type of the module, and E_ij_ represents the specific attribute parameters of the module. In the figure, there are 2 clothing nodes, 3 component module types, 5 specific module nodes, 4 attribute types, and 8 specific attribute parameters. For example, C1 and C2 are two types of T-shirts, and module types S1, S2, and S3 are the body module, sleeve module, and hem module, respectively. Then specific modules S11 and S12 in the body module can be H and X body module, respectively, and attribute variables E1, E2, E3, and E4 can be lapel, length, profile, and bottom, so E11 and E12 can be lapel or single breasted lapel, E21, E22, and E23 can be regular length, middle section, and long section, E31 and E32 are H and X, and E41 is straight bottom, respectively. Fuzzy ontology representation involving the uncertainty of the user and book concept evaluation is shown in Figure 2.

## 3. Genetic Clustering Subsystem

### 3.1. Different Degrees

Many clustering algorithms are based on the difference matrix for clustering analysis. The difference matrix is an object–object structure. It stores the differences between all *N* objects. It is generally represented by an *n∗n* matrix,

(1) $$\begin{array}{c} \left[\begin{array}{cccc} 0 & \; & \; & \;\\ d\left(2,1\right) & 0 & \; & \;\\ \ldots & \ldots & 0 & \;\\ d\left(n,1\right) & d\left(n,2\right) & \ldots & 0 \end{array}\right], \end{array}$$

where *d*(*i*, *j*) represents the degree of difference between object *i* and object *j*, which is used to measure the degree of difference between two objects. In general, *d*(*I*, *j*) is a non-negative number. When object *I* and object *J* are more similar or close to each other, the data are closer to 0, and the larger the value is, the less similar object *I* and object *J* are. In general, we have *d*(*i*, *j*) = *d*(*j*, *i*) and *d*(*i*, *i*) = 0.

Interval scale variable is a continuous variable of rough linear scale, such as length, mass, etc. The unit of the interval scale degree variable has a great influence on the clustering result, so it needs to be standardized first. Given the measure value of a variable *F*, the average absolute deviation *S*_*f*_ is calculated in the standardization process, as shown in formula (2):

(2) $$\begin{array}{c} s_{f}=\frac{1}{n}\left(\left|x_{1f}-m_{f}\left|+\right|x_{2f}-m_{f}\left|+\cdots +\right|x_{nf}-m_{f}\right|\right), \end{array}$$

where AAAA is the *n* measures of *f* and *m*_*f*_ is the average value of *f*, as shown in formula (3):

(3) $$\begin{array}{c} m_{f}=\frac{1}{n}\left(x_{1f}+x_{2f}+\cdots +x_{nf}\right). \end{array}$$

Then the standardized measurements are calculated, as shown in formula (4):

(4) $$\begin{array}{c} z_{if}=\frac{x_{if}-m_{f}}{s_{f}}. \end{array}$$

After normalization, or in some applications where normalization is not required, the dissimilarity between objects is calculated based on the distance between objects. The most commonly used distance formula is Euclidean distance, as shown in formula (5):

(5) $$\begin{array}{c} d\left(i,j\right)={\left({\left|x_{i1}-x_{j1}\right|}^{2}+{\left|x_{i2}-x_{j2}\right|}^{2}+\cdots +{\left|x_{ip}-x_{jp}\right|}^{2}\right)}^{1/2}, \end{array}$$

where *i*=(*x*_*i*1_+*x*_*i*2_+⋯+*x*_*ip*_) and *j*=(*x*_*j*1_+*x*_*j*2_+⋯+*x*_*jp*_) represent a *P*-dimensional data object.

Another commonly used distance calculation method is Manhattan distance, whose specific calculation formula is defined in formula (6):

(6) $$\begin{array}{c} d\left(i,j\right)=\left|x_{i1}-x_{j1}\right|+\left|x_{i2}-x_{j2}\right|+\cdots +\left|x_{ip}-x_{jp}\right|. \end{array}$$

Minkowski distance is a generalization of Euclidean distance and Manhattan distance, and its calculation formula is as shown in formula (7):

(7) $$\begin{array}{c} d\left(i,j\right)={\left({\left|x_{i1}-x_{j1}\right|}^{q}+{\left|x_{i2}-x_{j2}\right|}^{q}+\cdots +{\left|x_{ip}-x_{jp}\right|}^{q}\right)}^{1/q}. \end{array}$$

Here, *Q* is a positive integer. When *Q* = 1, it represents the Manhattan distance calculation formula; when *q* = 2, it represents the Euclidean distance calculation formula. If each variable can be assigned a weight to indicate the importance of the attribute it represents, the calculation formula of Euclidean distance with weight is as shown in formula (8):

(8) $$\begin{array}{c} d\left(i,j\right)={\left(\omega_{1}{\left|x_{i1}-x_{j1}\right|}^{2}+\omega_{2}{\left|x_{i2}-x_{j2}\right|}^{2}+\cdots +\omega_{p}{\left|x_{ip}-x_{jp}\right|}^{2}\right)}^{1/2}. \end{array}$$

Similarly, Manhattan distance and Minkowski distance can also be calculated by introducing weights.

## 4. Experimental Results and Analysis

**Figure 3:**
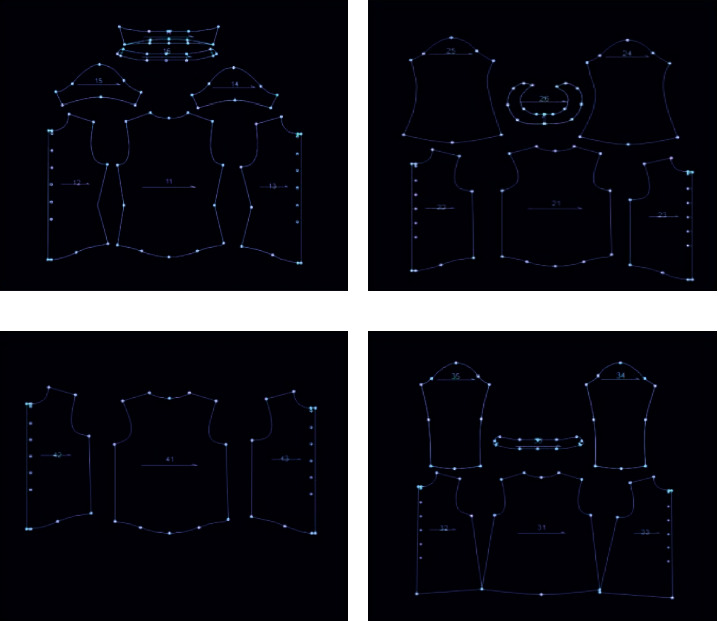
User interface.

The system first interacts with the user. A certain number of clothing pictures are listed for the user, and the user selects and scores the clothes suitable for him, as shown in Figure 3. The number of clothes selected by the user is determined by the user based on the “body-indented” style.

Twenty clothes were selected and scored. The chromosome codes and user scores are shown in Table 1.

The influence degree of collar, sleeve, garment piece, and waist on style is 40%, 25%, 25%, and L0%, respectively. The crossover probability is 0.7, the mutation probability is 0.01, and the evolutionary cutoff algebra is 500. The results are shown in Table 2.

The top 4 chromosomes are taken as the preference matrix of the preference model, and the preferred model is:

The system recommends clothes again according to the preference model for users; from the database of all the clothes, the recommend 20 clothes are rated by the user, and for a user, an average of 83.74 shows the satisfaction of the user by the system-recommended clothes.

**Figure 4:**
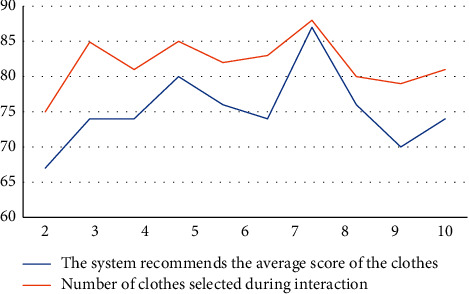
Analysis diagram of experimental results.

On a test of 10 users, the result is as shown in Figure 4. An average score of 82.671 in the test of 10 users on the system-recommended clothes shows that user satisfaction is high. In general, the more clothes the user chooses when interacting with the system, the higher the final score is, indicating that the more information the system obtains from the user, the closer the result is to the user's needs. An analysis diagram of the experimental results is shown in Figure 4.

It can be seen from Figure 4 that the genetic algorithm proposed in this paper has a greater advantage in the simulation results; the simulation accuracy is higher, and it is closer to the actual situation.

## 5. Conclusion

Collaborative filtering recommendation technology is the most successful recommendation technology, but there are two major problems, such as recommendation quality and scalability. Improving recommendation quality and improving scalability are inherently opposite to each other. At present, research at home and abroad mainly focuses on recommendation quality, and there is less discussion on scalability. Existing solutions to the scalability problem tend to reduce recommendation response time and recommendation quality. In this paper, the clustering analysis subsystem based on the genetic algorithm is innovatively introduced into the traditional collaborative filtering recommendation system, and its design and implementation are given. In addition, when obtaining the nearest neighbors, only the clustered users of the target user are searched, making it a collaborative filtering recommender system based on genetic clustering. The experimental results show that the response time of the traditional collaborative filtering recommender system increases linearly with the increase in the number of users while the response time of the collaborative filtering recommender system based on genetic clustering remains unchanged with the increase in the number of users. On the other hand, the recommendation quality of the collaborative filtering recommender system based on genetic clustering is basically not degraded compared with that of the traditional collaborative filtering recommender system. Therefore, the collaborative filtering recommender system based on genetic clustering can effectively solve the scalability problem of the collaborative filtering recommender system. However, for the scalability of the recommender system, there is still more content to be simulated in this study.

## Figures and Tables

**Table 1:** Coding and user ratings.

Clothing serial number	Chromosomal coding				User ratings
1	0000	0011	0000	1001	87
2	1110	1010	0000	0001	80
3	0111	0001	1010	1110	90
4	1010	1001	0101	0000	75
5	1001	11l1	0101	0111	85
6	0000	0011	0010	0100	81

**Table 2:** Final results of genetic algorithm.

The sorting	Chromosomal coding				Fitness
1	0000	0011	0100	1100	26.903
2	0000	1101	0110	0110	25.192
3	0101	0111	1001	0001	23.6
4	1010	1011	0101	1100	23.004
5	0110	1011	0100	0011	22.474
6	0000	0100	0001	0111	20.111
7	1001	0000	1101	0001	18.944
.	…				…
.	…				…
.	…				…

## Data Availability

The data used to support the findings of this study are available from the corresponding author upon request.
